# Value of gym-based group exercise versus usual care for young adults receiving antipsychotic medication: study protocol for the multicenter randomized controlled Vega trial

**DOI:** 10.1186/s12888-023-05086-z

**Published:** 2023-08-30

**Authors:** Bolette Skjødt Rafn, Martin Færch Andersen, Victor Sørensen, Eik Dybboe Bjerre, Lone Baandrup, Ditte Lammers Vernal, Ole Mors, Filip Krag Knop, Rasmus Trap Wolf, Anders Tolver, Joseph Firth, Nikolaj Nøhr, Søren T. Skou, Bjørn H. Ebdrup, Julie Midtgaard

**Affiliations:** 1 Center for Applied Research in Mental Health Care (CARMEN), Mental Health Center Glostrup, University of Copenhagen, Glostrup, Denmark; 2 Danish Cancer Society National Cancer Survivorship and Late Effects Research Center (CASTLE), Department of Oncology, Copenhagen University Hospital Rigshospitalet, Copenhagen, Denmark; 3 Department of Physiotherapy, University College of Northern Denmark, Aalborg, Denmark; 4 Center for Neuropsychiatric Schizophrenia Research (CNSR), Mental Health Centre Glostrup, University of Copenhagen, Copenhagen, Denmark; 5 Mental Health Centre Copenhagen, Copenhagen, Denmark; 6 Faculty of Health and Medical Sciences, Department of Clinical Medicine, University of Copenhagen, Copenhagen, Denmark; 7 Psychiatry, Aalborg University Hospital North, Aalborg, Denmark; 8 Department of Clinical Medicine, Aalborg University, Aalborg, Denmark; 9 Psychosis Research Unit, Aarhus University Hospital, Aarhus, Denmark; 10 Center for Clinical Metabolic Research, Gentofte Hospital, University of Copenhagen, Hellerup, Denmark; 11 Steno Diabetes Center Copenhagen, Herlev, Denmark; 12 Data Science Lab, Department of Mathematical Sciences, University of Copenhagen, Copenhagen, Denmark; 13 Division of Psychology and Mental Health, University of Manchester, Manchester Academic Health Science Centre, Manchester, UK; 14 Arca, Denmark; 15 Research Unit for Musculoskeletal Function and Physiotherapy, Department of Sports Science and Clinical Biomechanics, University of Southern Denmark, Odense, Denmark; 16 The Research Unit PROgrez, Department of Physiotherapy and Occupational Therapy, Næstved-Slagelse-Ringsted Hospitals, Slagelse, Region Zealand Denmark

**Keywords:** Physical activity, Severe mental illness, Recovery, High-intensity Functional Training, Antipsychotics, Community, Loneliness, Stigmatization

## Abstract

**Background:**

Exercise is recommended to protect physical health among people with severe mental illness and holds the potential to facilitate long-term recovery. An inclusive exercise community provides an opportunity for life skill training and social connectedness and may reduce the experience of loneliness and internalized stigmatization which together may improve personal recovery. Using a pragmatic randomized design, we aim to examine the effectiveness of a gym-based exercise intervention tailored to young adults in antipsychotic treatment (i.e., Vega Exercise Community) compared to usual care. It is hypothesized that the Vega Exercise Community will be superior to usual care for personal recovery at four months.

**Methods:**

The trial will be conducted at four sites in Denmark from which 400 participants, aged 18 to 35 years, who are in current treatment with antipsychotic medications for the management of schizophrenia spectrum or affective disorders, will be recruited. Participants will be randomized (2:1) to Vega Exercise Community or usual care. Vega Exercise Community includes three weekly group-based exercise sessions hosted in commercial functional training centers delivered by certified Vega instructors. After four months, participants in Vega Exercise Community will be randomized (1:1) to minimal versus extended support with regards to sustained physical activity. Data will be collected at baseline, four, six and 12 months. The primary outcome is personal recovery assessed by Questionnaire about the Process of Recovery at four months. Behavioral symptoms, health-related quality of life, metabolic health, and program costs will be evaluated to further determine the effectiveness and cost-effectiveness of the Vega Exercise Community. Finally, the quality of life and physical and mental health of the participants’ primary relative will be evaluated.

**Discussion:**

The results of this trial may have important implications for health, sustained physical activity, and recovery for individuals in treatment with antipsychotics. Given the pragmatic design, positive results may readily be implemented by mental health care professionals to promote exercise as an integrated part of treatment of severe mental illness.

**Trial registration:**

Clinical Trials.gov (NCT05461885, initial registration June 29th, 2022). WHO Universal Trial Number (UTN): U1111-1271–9928.

**Supplementary Information:**

The online version contains supplementary material available at 10.1186/s12888-023-05086-z.

## Background

Psychotic disorders are severe and enduring mental health conditions, which are often identified when people are in their young adulthood thus resulting in disruption to education, employment, and life prospects [1]. Specialized interventions that take place soon after the onset of the first episode of psychosis, and offered as an adjunct to treatment with antipsychotics, are associated with reduced symptoms and improved overall functioning [2–4]. Using exercise as part of early specialized interventions to treat negative and cognitive symptoms among young adults receiving antipsychotic medication holds the potential to facilitate long-term clinical recovery, as early improvements in these areas reduce the likelihood of enduring chronic symptoms and functional disability [1]. In addition to having beneficial effects on psychotic symptoms (i.e., clinical recovery) [5], negative and depressive symptoms, global functioning, and quality of life [6–9], exercise also improve multiple cardiometabolic outcomes [10, 11]. This is especially important bearing in mind the greatly increased risk for weight-gain and diabetes [12, 13] associated with antipsychotic treatment.

When building sustainable and engaging exercise routines, it is recommended to provide a combination of aerobic and strength training and a range of exercise options accommodating peoples’ preferences and goals [11]. Specifically, previous research indicate that gym-based activities were substantially more popular among people with psychosis than other sporting activities [14]. As such, group-based exercise hosted in commercial functional training centers (i.e., CrossFit™ centers), which incorporates functional movements that increase strength and cardiorespiratory fitness, can be a possible novel clinical treatment strategy for people with psychosis [11, 15]. Further, an exercise community offers social inclusion and support in addition to training in social skills and thus has the potential to reduce loneliness and internalized stigmatization [16] which is of importance to peoples’ personal recovery [17, 18]. Recovery is now among the most influential paradigms shaping mental health policy and practice [19, 20]. It poses an alternative to the biological view of mental illness which focus on alleviating symptoms. In contrast, the notion of “personal recovery” entails living a meaningful, satisfying, empowered, and hopeful life even if the symptoms of the mental illness persists [17]. Personal recovery is related to clinical recovery and thus recommended as a clinical endpoint for research interventions [21].

Our pilot trial of supervised, group-based exercise delivered in a commercial fitness center for people with first episode psychosis [22, 23] and other trials [24–29] suggest that exercise is feasible and meaningful to people and associated with positive changes in personal [22, 23] and clinical recovery [24, 25], and increased levels of physical activity [27], weight loss [26], and reduced cardiovascular risk [28]. However, because of strict inclusion criteria, the external validity of existing studies, including transferability of interventions into real-world settings, is limited, and meta-review authors conclude that effectiveness studies are urgently needed [6, 11].

While the central actor in personal recovery always is the person with mental illness, the notion of ‘‘family recovery’’ has recently gained currency among both researchers and mental health practitioners [30, 31]. Indeed family members, friends and other persons close to the patient play decisive roles in the recovery process and in the long-term delivery of effective treatment [32]. Specifically, relatives often take significant responsibility in managing the patient’s treatment appointments and medication [33]. However, caregiving of patients with severe mental illness imposes a substantial burden on the relatives [34, 35]. This burden include economic difficulties, negative effects on physical and mental health, impaired personal, social, and vocational capacity, family conflicts, separation, decreased quality of life, emotional distress, loss of self-esteem, and increased alcohol use [35]. Given the emphasis on family involvement in the recovery process of mental illness, it is important to understand what impact an exercise community designed to improve personal recovery for the patient may have on the relatives’ physical and mental health.

This paper presents the protocol of the Vega trial based in Denmark. The aim is to examine the effectiveness of an exercise intervention tailored to young adults in antipsychotic treatment delivered in a functional exercise environment (Vega Exercise Community) offered in addition to usual care and compared to usual care alone. We hypothesize that the Vega Exercise Community will be superior to usual care for personal recovery at four months.

## Methods

### Study design

The Vega trial will be a multi-center, pragmatic, randomized (2:1) trial comparing a four-month, supervised, gym-based group exercise program to usual care on changes in personal recovery. The pragmatic nature of the trial entails that it is designed to evaluate the effectiveness of intervention in real-life routine practice conditions [36, 37]. In order to explore different ways to support sustainment (i.e., post-intervention adoption of physical activity), and to guide strategies to facilitate adherence, we will do “a study within a trial” (SWAT) [38]. Hence participants randomized to the intervention at baseline, will be randomized (allocation ratio 1:1) after four months to minimal vs. extended support with regards to sustainment of physical activity. The protocol is reported according to the SPIRIT checklist (Supplementary file 1 and 2) [39].

**Fig. 1:**
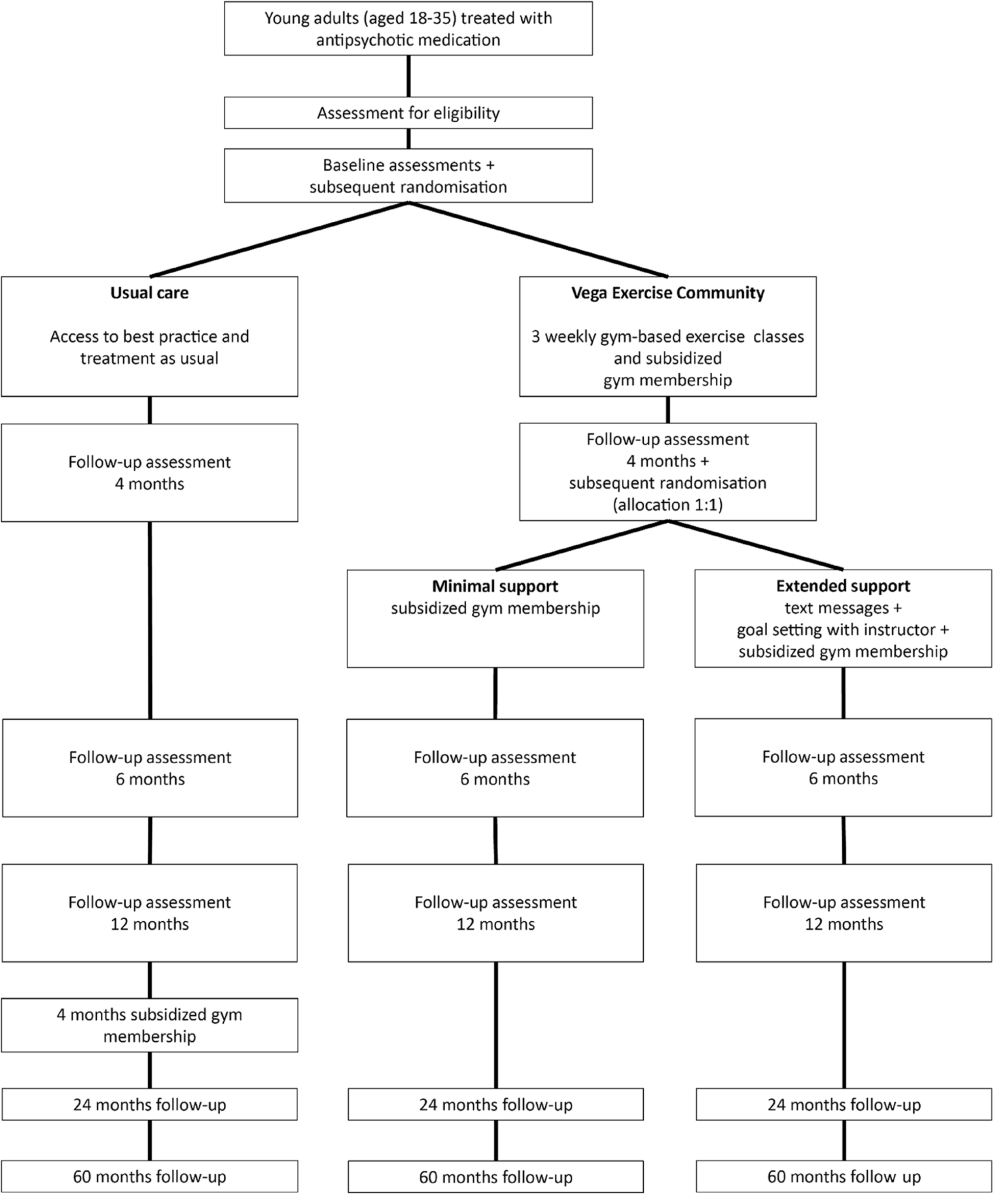
Flow chart

Participants allocated to the usual care group will be offered free-of-charge access to the Vega Exercise Community for the remaining duration of the study after completing the 12 months end of study visit. This is to minimize attrition and ensure that all enrolled participants are offered access to the exercise community. Similarly, participants allocated to the intervention group will be offered free-of-charge access to the Vega Exercise Community for the duration of the study. Finally, the long-term use of mental health care resources will be collected from medical records at 24 months and 60 months. The trial will recruit participants from the North Denmark Region, the Central Denmark Region and Capital Region of Denmark during an anticipated 24 months. The trial design is outlined in Fig. 1.

### Study aims

The primary aim is to examine the effectiveness of the Vega Exercise Community versus usual care for:

i) Improving personal recovery measured by the Questionnaire about the Process of Recovery at four months.

The secondary aims are examination of the effectiveness of Vega Exercise Community on:

ii) Mental health at four months (main secondary outcome measured by Short-Form-12 Mental Component Summary).
iii) Other health outcomes (health-related quality of life, behavioral symptoms, metabolic health) at four, six and twelve months.

Tertiary aims include investigation of:

iv) effect of prolongation of subsidized gym membership in addition to motivational text messages and individual meeting with instructor to set behavior goals (extended support) compared to subsidized gym membership alone (minimal support), and to treatment as usual in relation to post-intervention adoption of physical activity at six and twelve months;
v) the impact of the Vega Exercise Community on the quality of life and physical and mental health of the participants’ primary relative at four, six and twelve months;
vi) the cost-effectiveness of the Vega Exercise Community expressed as the incremental cost-effectiveness ratio of the cost (education of instructors, delivery of the exercise sessions) and quality adjusted life years (QALY) from the EuroQol-5 Domain questionnaire (EQ-5D-5L) at four months; and
vii) participants’ long-term use of mental health care services at two and five years.

### Participant eligibility

People aged 18–35 years who are currently treated with antipsychotic medication on a daily basis for at least the past one month (any dose and antipsychotic medicament) for the management of a schizophrenia spectrum disorder (F20-F29) or an affective disorder (F30-F39), who read and speak Danish and are able to give informed consent will be eligible. Those who are advised against participating in exercise activities by their treating physician doctor (i.e., current injuries or instable somatic comorbidity) will be excluded. Women who are or become pregnant during the study period may participate but will not be assessed for body composition using bioimpedance due to manufacturer's contraindications. The Vega Exercise Community is intended as an adjunctive therapy to usual care. Ongoing pharmacological, lifestyle or other treatments during the intervention period is therefore not an exclusion criterion.

### Recruitment

The primary recruitment strategy will be through the outpatient mental health services, OPUS (an intensive early-intervention program for people with first-episode psychosis) and flexible assertive community treatment (F-ACT) teams, in three regions in Denmark, namely the Capital Region, North Denmark Region, and Central Denmark Region. The first information about the study will be given by health care professionals during outpatient treatment visits at the mental health centers. People who are interested will be given written information for them to read at home. These people will then be contacted by telephone by the study team, who will explain the study in detail and confirm the person’s eligibility and send them detailed written information. The person will be advised to identify their primary relative and bring them to the baseline visit if they wish to. Strategies to ensure adequate recruitment include a recruitment barometer with monthly updates to each site, newsletters, and acknowledgements (i.e., chocolate) to health care professionals when sub-goals are reached (i.e., *n* = 30 for a site).

Recruitment also occurs through community-based advertising such as posters and flyers displayed around mental health community hubs, media releases, newspaper articles, radio interviews, community talks, internet (www.projektvega.dk), and social media (@projekt_vega). Interested individuals undergo eligibility screening conducted over the phone with the study team. Eligible individuals are invited to enroll in the study.

### Data collection

Baseline and four, six and 12-months assessments will take place in private rooms during an in-person visit at the treatment centers. Baseline measurements will be completed following written informed consent obtained by research assistants (RAs). RAs will administer questionnaires and measurements. If needed, RAs will assist participants in completion of questionnaires either by reading aloud the questions or by clarifying questions while the participant completes the questionnaire. All data will be collected through electronic data capture (REDCap®). During the visit, RAs will collect data on antipsychotic medications, and latest analyzed blood sample results from the medical records.

To promote data quality, two to three measurements of most metabolic outcomes are taken and the average recorded. A bioimpedance device is used to assess body composition (i.e., fat-free mass, fat mass, and skeletal muscle mass) based on the rate at which an electrical current travels through the body [40]. Cardiorespiratory fitness is measured with the revised Ekblom-Bak submaximal cycle ergometer test [41, 42]. RAs who will conduct the data collection will be thoroughly trained by the core research team (VS, MFA) and will be blinded to participants’ group allocation. Therefore, participants will be reminded prior to each follow-up assessment not to reveal the group they have been assigned to retain blinded status of the RAs. To promote retention, participants will receive a gift card of DKK100 (€13.50) per visit. All outcome data will be collected for participants who deviate from intervention protocol (i.e., those who do not meet the per protocol criteria for exercise attendance) while no outcome data will be collected for participants who discontinue the trial.

Finally, at 24 months and 60 months, use of inpatient and outpatient hospital mental health care services including contacts with mental health emergency departments will be obtained from the medical records to evaluate the long-term benefit of the program (i.e., reduced use of mental health care services).

### Outcome measures

A summary of outcome measures is displayed in Table 1. Schedule of enrolment, intervention, and assessments is displayed in Table 2. Personal recovery assessed by the 15-item Questionnaire about the Process of Recovery (QPR) [43] will be the primary outcome of the study. QPR was developed to measure recovery among people with psychosis and has been suggested to be a psychometrically sound instrument for measuring personal recovery among people with severe mental illness [43]. It assesses the level of agreement with various statements that the respondent has experienced in the past 7 days on a 5-point Likert scale ranging from 0 (strongly disagree) to 4 (strongly agree). Scores range from 0 to 60.

**Table 1:** Summary of assessments

**Endpoint**	**Domain**	**Measure**	**Instrument / Method**	**Data supplied by**
**Primary**	Recovery	Personal recovery	15-item Questionnaire about the Process of Recovery (QPR) [44]	Participant (rating self) *and *participant’s primary relative (rating participant)
**Main Secondary**	Mental health	Mental Component Summary (MCS)	12-item Short-Form-12 (SF-12) Health Survey [45]	Participant (rating self) *and* participant’s primary relative (rating self)
**Secondary**	Health-related quality of life	Physical Component Summary (PCS)	12-item Short-Form-12 (SF-12) Health Survey [45]	Participant (rating self) *and *participant’s primary relative (rating self)
		Physical role, bodily pain, general health, vitality, social functioning, emotional role		
	Behavioral symptoms	Affective symptoms	4-item Patient-Reported Outcomes Measurement Information System (PROMIS) Emotional distress (depression) [46]	Participant (rating self) *and* participant’s primary relative (rating self)
		Physical activity	7-item International Physical Activity Questionnaire short form (IPAQ-SF) [47]	
		Sleep	5-item Pittsburgh Sleep Quality Index (PSQI) [48]	
		Internalized Stigma of Mental Illness	9-item Internalized Stigma of Mental Illness Inventory (ISMI-9) [49]	Participant (rating self)
		Substance abuse	3-item Self-developed questionnaire	
		Positive and negative symptoms	14-item Modified Colorado Symptom Index (MCSI) [50]	
		Loneliness	Single-item measure	
	Metabolic health	Abdominal and hip circumference, weight, height, BMI	Anthropometry	Blinded assessor (rating participant)
		Total and visceral fat mass and muscle mass	Non-invasive bioimpedance analysis	
		Cardiorespiratory fitness	Ekblom-Bak submaximal cycle ergometer test	
		Blood pressure and resting heart rate	Digital blood pressure monitor	
		Glycosylated haemoglobin (HbA1c)	Blood samples and biochemical analysis	Routine blood samples
		Blood lipids (total cholesterol, high density lipoproteins, triglycerides)		
**Tertiary**	Cost-effectiveness	Health care usage	Inpatient and outpatient hospital care	Medical records
		Quality of life	5-item Euroqol EQ-5D-5L [51, 52]	Participant (rating self)

**Table 2:**
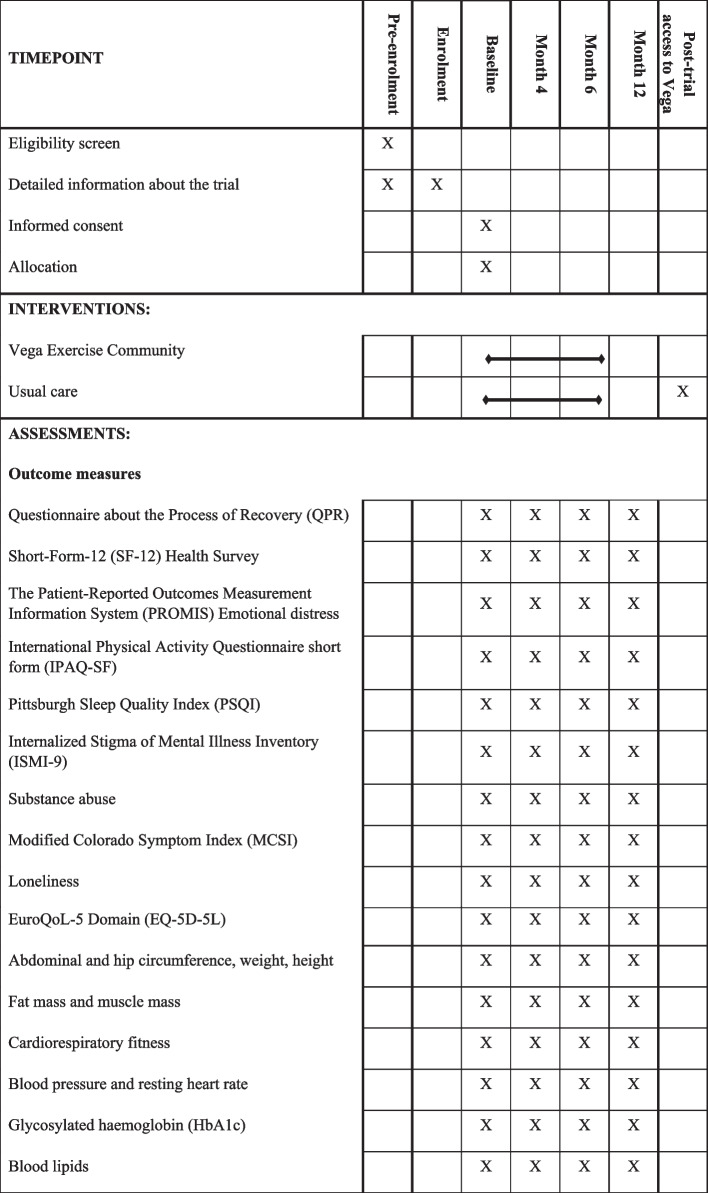
Schedule of enrolment, intervention, and assessments

The main secondary outcome is the Short-Form-12 Mental Component Summary, while other secondary outcome measures were chosen as they assess various relevant aspects of physical health and well-being including symptoms, sleep, depression, fitness, and health-related quality of life.

### Safety

Procedural safety measures will be in place for participants exhibiting deterioration in mental health, such as signs of suicidality, an adverse event related to the exercise intervention, or elevation in positive and negative symptoms. As this is a non-drug trial, using interventions that are within recommended exercise guidelines, the risk of severe adverse events (SAEs) related to the exercise intervention is low. An adverse event (AE) can be any adverse or unintended reaction, every symptom no matter whether causality with the intervention is likely. The exercise intensity and complexity will be adjusted to individual needs to minimize the risk of AEs. Data on SAEs and AEs will be collected by the instructors.

In addition to collection of AE and SAE in relation to the delivery of the intervention, the research team will collect information from the medical record system when a participant, regardless of group, is admitted or has contact with the emergency room due to a mental health issue. Finally, participants complete a questionnaire on SAEs and AEs and the Modified Colorado Symptom Index (MCSI) at every study visit, which is overseen by the study coordinator and medical personnel. MCSI consists of 14 items to measure positive and negative symptoms [53]. Reports on the MCSI of thoughts about self-harm, suicidal behavior or harm to others will initiate safety protocol procedures.

### Vega exercise community

The Vega Exercise Community program has been developed by exercise physiologists, physical therapists, and a consultant (NN) with expertise in developing functional exercise programs with the goal to support recovery for mental health [54, 55]. Vega Exercise Community will be hosted at commercial functional gyms and consists of one hour of supervised group exercise sessions offered three times per week for four months and will be offered in addition to usual care. In addition, participants will be offered free-of-charge membership to the gym from baseline to six months follow-up and are invited to take part in exercise sessions and use fitness equipment provided by the gym to regular members. The supervised program will be tailored to meet the needs and requirements of this group of young adults and include moderate-to-high intensity and mobility exercises. Two experienced instructors recruited from the local functional gym will supervise all exercise sessions.

The exercise sessions will start with warm-up exercises, followed by 10–20 min resistance training (Part A), 10–20 min High Intensity Functional Training (HIFT) (Part B), and cool down. Instructors will be allowed to make adjustments as the delivery of the intervention is pragmatic [36, 37]. Of importance, participants will be allowed to either perform the exercise together as a group, in pairs or one-by-one depending on needs and preferences. Participants will be invited to bring a friend or relative as a “plus one” to participate in the exercise session free-of-charge. Also, peer mentors will be associated with each gym and invited to participate free-of-charge alongside participants allowing the possibility of have constructive dialogues with instructors about optimizing the exercise community and support for participants*.* A detailed description of the intervention according to the TIDieR template is provided in Supplementary file 3.

### Strategies to increase exercise self-efficacy and adherence

In accordance with social cognitive theory [56], the intervention will offer exercise in groups allowing for the possibility of social modelling and/or vicarious experiences. Instructors and peer mentors will focus on means to aid participants’ experience of mastery (e.g., emphasizing and promoting the value of a strong body rather than a slim body). To further support development of exercise self-efficacy, instructors will meet with each participant individually before initiation of the exercise program. During this meeting, the participant will complete a few exercise self-efficacy items to be used by the instructors to explore the participant’s previous exercise experiences and preferences and how the participant will prefer to be coached and receive feedback. This meeting will furthermore include dialogue about development of individual behavioral goals. Instructors will send short text messages to participants prior to each exercise session encouraging them to show up. Strategies to support participants who repetitively miss sessions include additional support from peer mentors (i.e., identify the specific barriers and work with the participant to minimize those). Finally, participants will be encouraged to suggest exercises and music, and invited to socialize after each exercise session. The program theory is outlined in Supplementary file 4.

### Intervention adherence and fidelity

Participants’ attendance will be recorded by the instructors. Intervention fidelity will be measured by collecting data on intensity, duration, structure, and organization of the exercise sessions. Specifically, the duration, structure (i.e., warm up, resistance training, HIFT, and cooldown) and organization (i.e., presence of two instructors and peer mentor) will be reported by the instructors after each session. The exercise intensity will be monitored with heart rate monitors worn by the participants and self-reported using the Borg Scale of perceived exertion [57] in ten randomly selected sessions for each site.

### Minimal versus extended support for post-intervention sustainment of physical activity

After completion of the four-month intervention period, participants will be randomized into one of two strategies supporting sustainment of physical activity. One group will be offered prolonged free-of-charge gym membership *and* invitation to meet the instructor individually once to discuss behavior goals for the coming two months *and* receive reminder short text messages prior to each exercise session (i.e., extended support) for a period of two months. In addition, the peer mentor will support this group in attending regular exercise sessions at the gym and in attending other physical activities if preferred. The other group will be offered prolonged free-of-charge subsidized gym membership only (i.e., minimal support), also for a period of two months.

### Usual care

Participants allocated to the usual care group will receive treatment as usual and be informed of the official physical activity guidelines as part of the information on group allocation. Moreover, they are advised to continue their daily living, not guiding them to other interventions neither preventing them to do so. In addition, participants in the usual care group will be given a subsidized membership including access to the Vega Exercise Community program for four months after the 12 months follow-up.

### Allocation and blinding

All people who meet the inclusion criteria and provide written informed consent will be randomized during the in-person visit after baseline assessments have been completed. Participants will be assigned to Vega Exercise Community or usual care using an allocation ratio of 2:1.

A block randomization list, stratified by center and diagnosis (F20/F30), with varying block sizes will be used. The list will be developed using the R-package “blockrand” [58] (R Core Team, 2022) with a unique matrix-generating number ensuring blinding of the developer (VS) and implemented using the REDCap® system, which conceal the forthcoming allocation to all staff working on the trial.

After four months, participants allocated to the intervention group at baseline will be randomized, stratified to center and diagnosis (F20/F30), to either minimal or extended support, in a similar manner as the randomization at baseline.

Blinding of participants, relatives, or instructors will not be possible due to the nature of the trial. However, baseline and follow-up assessments will be undertaken by RAs blinded to allocation. Researchers who undertake data management and analysis will likewise be unaware of allocation.

### Sample size

The sample size was chosen to yield sufficient power for detection of a difference of 5 points between the two groups on the primary outcome measuring change from baseline to four months in QPR. No previous studies have reported the SD on change scores, nor minimal clinical important difference, based on the 15-item version of QPR, but experience with similar questionnaires indicate that SD for raw scores and changes are comparable. We therefore used a standard deviation (SD) of 13.8 points which corresponds to an effect size of 0.36 based on a previous trial [59] comparing the effects of a recovery-oriented intervention versus traditional services on personal recovery in people with mental illness using the 22-item version of QPR (SD 16.2). Considering an allocation ratio of 2:1 in favor of the intervention group, a two-sided t-test with a significance level of 5% yields a power of 83% if 200 participants are included in the intervention group and 100 in the usual care group. We anticipate drop-out (i.e., participants are lost to four-month follow-up) of 20% and therefore a minimum of 375 participants is needed. However, given the limited research utilizing the QPR, we aim to recruit 400 participants. This sample size will allow the detection of a clinically relevant difference between groups on our main secondary outcome, the Short-Form-12 Mental Component Summary, of four points at four-months with a SD of 12 points. The SD is based on a previous trial [60] comparing the effects of first-generation antipsychotics versus second-generation antipsychotics on quality of life in people with schizophrenia. No interim analysis will be performed. If 400 participants have not been enrolled by December 2024, recruitment will end, and final analyzes will be performed with the number of participants recruited at that point.

Because anchor-based estimations of minimal clinical important difference (MCID) on QPR are currently not available, we will supplement the data collection with one question on perceived improvement as an external criterion (“anchor”), i.e. asking participants (on a Likert scale of 7 levels) how they perceive a change (improvement or worsening) from baseline or last follow-up (1, worse than ever; 2, much worsened; 3, slightly worsened, 4, unchanged, 5, slightly improved; 6, much improved; 7, completely improved) and calculate an empirical derived anchor-based MCID [51].

### Data analysis

The main analysis will be based on the intention-to-treat principle where patients are analyzed according to the group to which they were randomly allocated. The primary outcome (personal recovery from QPR) and numerical secondary and tertiary outcomes will be analysed using a mixed effects model including relevant covariates (e.g., stratification variables) as well as main and interaction effects of assessment time and treatment as fixed effects. Random effects and appropriate correlation structures (depending on the number of assessment times to be modeled) will be included to capture the within subject dependence. Per protocol analyzes will be performed to estimate the de jure effect of the intervention. The per protocol population is defined as intervention group participants who attended at least 16 (33%) of the exercise sessions during the four-month period. The per-protocol analyzes will be adjusted with baseline covariates on disease and lifestyle characteristics to adjust for confounding variables that cannot be stratified for and that may arise when a sub-group (the per-protocol population) is analyzed [44]. Treatment effects will be quantified and interpreted using confidence intervals and significance tests on a 5% significance level. No formal adjustment of p-values will be applied, but the explorative nature and risk of false positive findings for secondary outcomes will be carefully addressed when results are presented. A subsequent within-trial health economic evaluation will be conducted from the service delivery perspective by including intervention costs (specifically cost of education course for instructors, salary to instructors and peer mentors, cost of exercise equipment etc. related to delivery of intervention), compared to quality-adjusted life years calculated from EuroQol-5 Domain (EQ-5D-5L) questionnaire [50, 52] combined with preference weights of the general Danish population [61].

### Ethics and dissemination

Approval to conduct the study was received from The Ethics Committee for the Capital Region of Denmark (H-21079211) and the Danish Data Protection Agency (P-2022–78). The trial is registered at clinicaltrials.gov (NCT05461885). Important protocol modifications will be documented in protocol amendments, which must be approved by the Ethics Committee for the Capital Region of Denmark and reported when the study is disseminated. The study will be conducted according to the Declaration of Helsinki (2008) and the International Conference on Harmonisation – Good Clinical Practice (ICH-GCP). As this is a non-drug trial, data monitoring and auditing is not warranted. However, the core research team (BSR, MFA, VS) monitor all aspects of the trial continuously including recruitment rate for each site, parameters related to the delivery of the intervention, attendance by participants to the intervention and assessments etc. Individual, electronic consent will occur prior to any testing procedures taking place. As the exercise intervention is designed to be an adjunctive therapy, all participants will be advised to continue usual care from their treating clinician while participating in the study. In accordance with the Declaration of Helsinki Dissemination Policy, research findings will be disseminated as widely as possible including an open access repository, conferences proceedings, presentations, and peer reviewed journals. To protect confidentiality of potential and enrolled participants, data access groups will be employed in REDCap® ensuring that RAs only access records for the particular site. The sponsor (JM), principal investigator (BSR), and core research team (VS, MFA) will have access to the final trial dataset, and statisticians (AT, RTW) will have access to pseudo-anonymized datasets. Findings will be reported using the Consolidated Standards of Reporting Trials (CONSORT) statement [62].

## Discussion

The cornerstone for treating psychotic disorders is second-generation antipsychotics, and a substantial proportion of the prescriptions for continuous antipsychotic medication are commenced when people are in their late teens or early 20 s. While these medications can be effective for reducing psychotic symptoms, they also confer a myriad of metabolic side-effects [12, 13] for which exercise and other life style programs are demonstrated beneficial [26–29]. Despite clinical guideline recommendations that lifestyle-based approaches (particularly physical activity and exercise) should be a foundational component of mental health care [63] and first line approach for protecting physical and mental health [11], this is seldomly delivered as part of mainstream clinical practice. The Vega Exercise Community provides an opportunity for young adults to access an exercise environment that is equally challenging and caring and support the integration of being a young individual along with the experience of mental illness [23]. Further, the Vega trial is the first to examine the effectiveness of an exercise intervention on personal recovery and also first to generate knowledge about and involve the primary relatives of the participants.

If the Vega Exercise Community program is superior to usual care in improving recovery and health, it has the potential not only to provide supplement care to individuals with mental illness but also enable mental health care professionals to promote exercise as an integrated part of treatment of severe mental illness. This trial will provide the first real-world data on the effectiveness and cost-effectiveness of exercise in commercial functional gym environments compared to usual care. It is anticipated that the intervention will produce dual mental and physical health benefits that may improve long-term health and result in cost-savings.

### Supplementary Information

**Additional file 1: **WHO Trial Registration Data Set.

**Additional file 2: **SPIRIT Checklist.

**Additional file 3: **The TIDieR (Template for Intervention Description and Replication) Checklist.

**Additional file 4: **Vega Program theory.

## Data Availability

Data sharing is not applicable to this article as no datasets were generated or analysed yet in the current study.

## Abbreviations

AEs: Adverse events
CONSORT: Consolidated Standards of Reporting Trials
EQ-5D-5L: EuroQol-5 Domain questionnaire
HIFT: High Intensity Functional Training
ICH-GCP: International Conference on Harmonisation – Good Clinical Practice
IPAQ-SF: International Physical Activity Questionnaire short form
ISMI-9: Internalized Stigma of Mental Illness Inventory
MCID: Minimal clinical important difference
MCS: Mental Component Summary
MCSI: Modified Colorado Symptom Index
PCS: Physical Component Summary
PROMIS: Patient-Reported Outcomes Measurement Information System
PSQI: Pittsburgh Sleep Quality Index
QPR: Questionnaire about the Process of Recovery
RAs: Research assistants
REDCap: Electronic data capture
SD: Standard deviation
SAEs: Severe adverse events
SF-12: Short-Form-12 Health Survey

## References

[1] Álvarez-Jiménez M, Gleeson JF, Henry LP, Harrigan SM, Harris MG, Killackey E, et al. Road to full recovery: longitudinal relationship between symptomatic remission and psychosocial recovery in first-episode psychosis over 7.5 years. Psychol Med. 2012;42(3):595–606. 10.1017/S0033291711001504 21854682

[2] Nordentoft M, Rasmussen JO, Melau M, Hjorthøj CR, Thorup AAE (2014). How successful are first episode programs? A review of the evidence for specialized assertive early intervention. Curr Opin Psychiatry maj.

[3] Albert N, Melau M, Jensen H, Emborg C, Jepsen JRM, Fagerlund B, et al. Five years of specialised early intervention versus two years of specialised early intervention followed by three years of standard treatment for patients with a first episode psychosis: randomised, superiority, parallel group trial in Denmark (OPUS II). BMJ. 2017;356:i6681. 10.1136/bmj.i6681 PMC5228538 28082379

[4] Marshall M, Rathbone J. Early intervention for psychosis. Cochrane Database Syst Rev. 2011;(6):CD004718. 10.1002/14651858.CD004718.pub3. 10.1002/14651858.CD004718.pub3 PMC4163966 21678345

[5] Michalska da Rocha B, Rhodes S, Vasilopoulou E, Hutton P (2018). Loneliness in Psychosis: A Meta-analytical Review. Schizophr Bull.

[6] Vancampfort D, Firth J, Correll CU, Solmi M, Siskind D, De Hert M, et al. The impact of pharmacological and non-pharmacological interventions to improve physical health outcomes in people with schizophrenia: a meta-review of meta-analyses of randomized controlled trials. World Psychiatr. 2019;18(1):53–66. 10.1002/wps.20614 PMC6313230 30600626

[7] Dauwan M, Begemann MJH, Heringa SM, Sommer IE (2016). Exercise Improves Clinical Symptoms, Quality of Life, Global Functioning, and Depression in Schizophrenia: A Systematic Review and Meta-analysis. Schizophr Bull maj.

[8] Sabe M, Kaiser S, Sentissi O. Physical exercise for negative symptoms of schizophrenia: Systematic review of randomized controlled trials and meta-analysis. Gen Hosp Psychiatry. 2020;62:13–20. 10.1016/j.genhosppsych.2019.11.002 31751931

[9] Bueno-Antequera J, Munguía-Izquierdo D (2020). Exercise and Schizophrenia. Adv Exp Med Biol.

[10] Ashdown-Franks G, Firth J, Carney R, Carvalho AF, Hallgren M, Koyanagi A, et al. Exercise as medicine for mental and substance use disorders: a Meta-review of the benefits for neuropsychiatric and cognitive outcomes. Sports Med. 2020;50(1):151–70. 10.1007/s40279-019-01187-6 31541410

[11] Firth J, Siddiqi N, Koyanagi A, Siskind D, Rosenbaum S, Galletly C, et al. The Lancet Psychiatry Commission: a blueprint for protecting physical health in people with mental illness. Lancet Psychiatry. 2019;6(8):675–712. 10.1016/S2215-0366(19)30132-4 31324560

[12] Schmitt A, Maurus I, Rossner MJ, Röh A, Lembeck M, von Wilmsdorff M, et al. Effects of aerobic exercise on metabolic syndrome, cardiorespiratory fitness, and symptoms in Schizophrenia include decreased mortality. Front Psychiatry. 2018;9:690. 10.3389/fpsyt.2018.00690 PMC6308154 30622486

[13] Krane-Gartiser K, Breum L, Glümrr C, Linneberg A, Madsen M, Køster A, et al. Prevalence of the metabolic syndrome in Danish psychiatric outpatients treated with antipsychotics. Nord J Psychiatry. 2011;65(5):345–52. 10.3109/08039488.2011.565799 21428861

[14] Firth J, Rosenbaum S, Stubbs B, Vancampfort D, Carney R, Yung AR (2016). Preferences and motivations for exercise in early psychosis. Acta Psychiatr Scand juli.

[15] Firth J, Rosenbaum S, Stubbs B, Gorczynski P, Yung AR, Vancampfort D (2016). Motivating factors and barriers towards exercise in severe mental illness: a systematic review and meta-analysis. Psychol Med.

[16] Yanos PT, DeLuca JS, Roe D, Lysaker PH (2020). The impact of illness identity on recovery from severe mental illness: A review of the evidence. Psychiatry Res juni.

[17] Anthony WA (1993). Recovery from mental illness: The guiding vision of the mental health service system in the 1990s. Psychosocial Rehabilitation J.

[18] Brooke LE, Lin A, Ntoumanis N, Gucciardi DF (2019). Is sport an untapped resource for recovery from first episode psychosis? A narrative review and call to action. Early Interv Psychiatry.

[19] Slade M, Amering M, Farkas M, Hamilton B, O’Hagan M, Panther G, et al. Uses and abuses of recovery: implementing recovery-oriented practices in mental health systems. World Psychiatry. 2014;13(1):12–20. 10.1002/wps.20084 PMC3918008 24497237

[20] Braslow JT (2013). The manufacture of recovery. Annu Rev Clin Psychol.

[21] Winsper C, Crawford-Docherty A, Weich S, Fenton SJ, Singh SP (2020). How do recovery-oriented interventions contribute to personal mental health recovery? A systematic review and logic model. Clin Psychol Rev.

[22] Midtgaard J, Schnor H, Bjerre ED, Jespersen T, Jelsøe N, Frølund N, et al. Exercise training complementary to specialised early intervention in patients with first-episode psychosis: a feasibility randomised trial. Pilot Feasibility Stud. 2021;7(1):162. 10.1186/s40814-021-00900-5 PMC8375206 34412705

[23] Larsen LQ, Schnor H, Tersbøl BP, Ebdrup BH, Nordsborg NB, Midtgaard J (2019). The impact of exercise training complementary to early intervention in patients with first-episode psychosis: a qualitative sub-study from a randomized controlled feasibility trial. BMC Psychiatry..

[24] Fisher E, Wood SJ, Upthegrove R, Aldred S (2020). Designing a feasible exercise intervention in first-episode psychosis: Exercise quality, engagement and effect. Psychiatry Res..

[25] Fisher E, Wood SJ, Elsworthy RJ, Upthegrove R, Aldred S (2020). Exercise as a protective mechanism against the negative effects of oxidative stress in first-episode psychosis: a biomarker-led study. Transl Psychiatry..

[26] Daumit GL, Dickerson FB, Wang NY, Dalcin A, Jerome GJ, Anderson CAM, et al. A behavioral weight-loss intervention in persons with serious mental illness. N Engl J Med. 2013;368(17):1594–602. 10.1056/NEJMoa1214530 PMC3743095 23517118

[27] Masa-Font R, Fernández-San-Martín MI, Martín López LM, Alba Muñoz AM, Oller Canet S, Martín Royo J, et al. The effectiveness of a program of physical activity and diet to modify cardiovascular risk factors in patients with severe mental illness after 3-month follow-up: CAPiCOR randomized clinical trial. Eur Psychiatry. 2015;30(8):1028–36. 10.1016/j.eurpsy.2015.09.006 26521223

[28] Bartels SJ, Pratt SI, Aschbrenner KA, Barre LK, Naslund JA, Wolfe R, et al. Pragmatic replication trial of health promotion coaching for obesity in serious mental illness and maintenance of outcomes. Am J Psychiatry. 2015;172(4):344–52. 10.1176/appi.ajp.2014.14030357 PMC4537796 25827032

[29] Green CA, Yarborough BJH, Leo MC, Yarborough MT, Stumbo SP, Janoff SL, et al. The STRIDE weight loss and lifestyle intervention for individuals taking antipsychotic medications: a randomized trial. Am J Psychiatry. 2015;172(1):71–81. 10.1176/appi.ajp.2014.14020173 PMC4282602 25219423

[30] Maybery D, Meadows G, Clark J, Sutton K, Reupert A, Nicholson J. A personal recovery model for parents with mental health problems. In A. Reupert, D. Maybery, J. Nicholson, M. Göpfert, & M. Seeman. eds. Parental Psychiatric Disorder: Distressed Parents and their Families. Cambridge: Cambridge University Press. 2015. pp. 312-323. 10.1017/CBO9781107707559.030.

[31] Seeman M. Creating Options for Family Recovery: A Provider’s Guide for Promoting Parental Mental Health. Book Review. Adv Ment Health. 2015;13:168-9. 10.1080/18387357.2015.1066292.

[32] Psychosis and schizophrenia in adults: prevention and management. NICE Clinical Guideline. 2014;39. Available at: https://www.nice.org.uk/guidance/cg178/resources/psychosis-and-schizophrenia-in-adults-prevention-and-management-pdf-35109758952133.

[33] Al-HadiHasan A, Callaghan P, Lymn JS (2017). Qualitative process evaluation of a psycho-educational intervention targeted at people diagnosed with schizophrenia and their primary caregivers in Jordan. BMC Psychiatry..

[34] Marquez JA, Ramírez García JI (2011). Family Caregivers’ Monitoring of Medication Usage: A Qualitative Study of Mexican-Origin Families with Serious Mental Illness. Cult Med Psychiatry.

[35] Awad AG, Voruganti LNP (2008). The burden of schizophrenia on caregivers: a review. Pharmacoeconomics.

[36] Loudon K, Zwarenstein M, Sullivan F, Donnan P, Treweek S (2013). Making clinical trials more relevant: improving and validating the PRECIS tool for matching trial design decisions to trial purpose. Trials..

[37] Bjerre E, Brasso K, Midtgaard J (2015). Pragmatic trials are important to medical research. Ugeskr Laeger..

[38] Treweek S, Bevan S, Bower P, Campbell M, Christie J, Clarke M, et al. Trial Forge Guidance 1: what is a Study Within A Trial (SWAT)? Trials. 2018;19(1):139. 10.1186/s13063-018-2535-5 PMC5824570 29475444

[39] Chan AW, Tetzlaff JM, Altman DG, Laupacis A, Gøtzsche PC, Krleža-Jerić K, et al. SPIRIT 2013 statement: defining standard protocol items for clinical trials. Ann Intern Med. 2013;158(3):200–7. 10.7326/0003-4819-158-3-201302050-00583 PMC5114123 23295957

[40] Khalil SF, Mohktar MS, Ibrahim F (2014). The Theory and Fundamentals of Bioimpedance Analysis in Clinical Status Monitoring and Diagnosis of Diseases. Sensors (Basel)..

[41] Ekblom-Bak E, Björkman F, Hellenius ML, Ekblom B. A new submaximal cycle ergometer test for prediction of VO2max. Scand J Med Sci Sports. 2014;24(2):319–26. 10.1111/sms.12014 23126417

[42] Björkman F, Ekblom-Bak E, Ekblom Ö, Ekblom B. Validity of the revised Ekblom Bak cycle ergometer test in adults. Eur J Appl Physiol. 2016;116(9):1627–38. 10.1007/s00421-016-3412-0 PMC4983286 27311582

[43] Law H, Neil ST, Dunn G, Morrison AP (2014). Psychometric properties of the questionnaire about the process of recovery (QPR). Schizophr Res juli.

[44] Hernán MA, Hernández-Díaz S, Robins JM. Randomized trials analyzed as observational studies. Ann Intern Med. 2013;159(8):560–2. 10.7326/0003-4819-159-8-201310150-00709 PMC3860874 24018844

[45] Jenkinson C, Layte R (1997). Development and testing of the UK SF-12 (short form health survey). J Health Serv Res Policy januar.

[46] Pilkonis PA, Yu L, Dodds NE, Johnston KL, Maihoefer CC, Lawrence SM (2014). Validation of the depression item bank from the Patient-Reported Outcomes Measurement Information System (PROMIS) in a three-month observational study. J Psychiatr Res september.

[47] Lee PH, Macfarlane DJ, Lam TH, Stewart SM (2011). Validity of the International Physical Activity Questionnaire Short Form (IPAQ-SF): a systematic review. Int J Behav Nutr Phys Act..

[48] Sancho-Domingo C, Carballo JL, Coloma-Carmona A, Buysse DJ (2021). Brief version of the Pittsburgh Sleep Quality Index (B-PSQI) and measurement invariance across gender and age in a population-based sample. Psychol Assess februar.

[49] Hammer JH, Toland MD (2017). Internal structure and reliability of the Internalized Stigma of Mental Illness Scale (ISMI-29) and Brief Versions (ISMI-10, ISMI-9) among Americans with depression. Stigma and Health.

[50] Mulhern B, Mukuria C, Barkham M, Knapp M, Byford S, Soeteman D. Using generic preference-based measures in mental health: psychometric validity of the EQ-5D and SF-6D. Br J Psychiatry. 2014;205(3):236–43. 10.1192/bjp.bp.112.122283 24855127

[51] Crosby RD, Kolotkin RL, Williams GR (2003). Defining clinically meaningful change in health-related quality of life. J Clin Epidemiol maj.

[52] König HH, Roick C, Angermeyer MC (2007). Validity of the EQ-5D in assessing and valuing health status in patients with schizophrenic, schizotypal or delusional disorders. Eur Psychiatry.

[53] Conrad KJ, Yagelka JR, Matters MD, Rich AR, Williams V, Buchanan M (2001). Reliability and validity of a modified Colorado Symptom Index in a national homeless sample. Ment Health Serv Res september.

[54] MF Andersen, K Roed, A Riis, BS Rafn, BH Ebdrup, J Midtgaard. Perspectives of professional experts in relation to the development of community-based exercise for young adults with schizophrenia – A qualitative study. medRxiv preprint. 10.1101/2023.08.03.23293592. 10.1136/bmjsem-2023-001658 PMC10533806 37780132

[55] MF Andersen, K Roed, V Sørensen, A Riis, BS Rafn, BH Ebdrup, J Midtgaard. What should be included in an educational programme for non-health professional exercise instructors in charge of community-based exercise targeting young adults in antipsychotic treatment – A focus group study of stakeholder perspectives. medRxiv preprint. 10.1101/2023.08.03.23293602.

[56] Bandura A (2004). Health Promotion by Social Cognitive Means. Health Educ  Behav..

[57] Borg GA.V (1982). Psychophysical bases of perceived exertion. Med Sci  Sports Exercise.

[58] Snow G. _blockrand: Randomization for Block Random Clinical Trials_. R package version 1.5. 2020. https://www.CRAN.R-project.org/package=blockrand.

[59] Meadows G, Brophy L, Shawyer F, Enticott JC, Fossey E, Thornton CD, et al. REFOCUS-PULSAR recovery-oriented practice training in specialist mental health care: a stepped-wedge cluster randomised controlled trial. Lancet Psychiatry. 2019;6(2):103–14. 10.1016/S2215-0366(18)30429-2 30635177

[60] Gründer G, Heinze M, Cordes J, Mühlbauer B, Juckel G, Schulz C, et al. Effects of first-generation antipsychotics versus second-generation antipsychotics on quality of life in schizophrenia: a double-blind, randomised study. Lancet Psychiatry. 2016;3(8):717–29. 10.1016/S2215-0366(16)00085-7 27265548

[61] Jensen CE, Sørensen SS, Gudex C, Jensen MB, Pedersen KM, Ehlers LH (2021). The Danish EQ-5D-5L Value Set: A Hybrid Model Using cTTO and DCE Data. Appl Health Econ Health Policy juli.

[62] Schulz KF, Altman DG, Moher D, CONSORT Group (2011). CONSORT 2010 statement: updated guidelines for reporting parallel group randomised trials. Int J Surg.

[63] Marx W, Manger SH, Blencowe M, Murray G, Ho FYY, Lawn S, et al. Clinical guidelines for the use of lifestyle-based mental health care in major depressive disorder: World Federation of Societies for Biological Psychiatry (WFSBP) and Australasian Society of Lifestyle Medicine (ASLM) taskforce. World J Biol Psychiatry. 2022;0(0):1–5. 10.1080/15622975.2022.2112074 PMC10972571 36202135

