# Supplementary material for: Value of gym-based group exercise versus usual care for young adults receiving antipsychotic medication: study protocol for the multicenter randomized controlled Vega trial
Source: BMC Psychiatry. 2023 Aug 30;23:634. doi: 10.1186/s12888-023-05086-z (PMC10466717; doi:10.1186/s12888-023-05086-z)
Supplement: Supplementary file 1 — Additional file 1: WHO Trial Registration Data Set. [file 12888_2023_5086_MOESM1_ESM.docx]

**Supplementary file 1: WHO Trial Registration Data Set**

1. **Trial registration:** ClinicalTrials.gov NCT05461885
2. **Registration** **date**: June 29^th^, 2022
3. **WHO Universal Trial Number** (UTN): U1111-1271-9928
4. **Funding:** TrygFonden (grant number 151603), Helsefonden (grant number 20-B-0328), and Toyota Foundation (grant number KJ/BG-10204 F).
5. **Sponsor:** Prof. Julie Midtgaard
6. **Secondary Sponsor(s):** None
7. **Contact for Public Queries**: Bolette Skjødt Rafn, PhD, Blegdamsvej 58, 2100 Copenhagen, +454015 6282, [bolette.skjoedt.rafn@regionh.dk](mailto:bolette.skjoedt.rafn@regionh.dk) or Prof. Julie Midtgaard, Nordstjernevej 41, 2600 Glostrup, Julie.klausen.midtgaard@regionh.dk
8. **Contact for Scientific Queries:** Bolette Skjødt Rafn, PhD, Blegdamsvej 58, 2100 Copenhagen, +454015 6282, bolette.skjoedt.rafn@regionh.dk or Prof. Julie Midtgaard, Nordstjernevej 41, 2600 Glostrup, Julie.klausen.midtgaard@regionh.dk
9. **Public title:** The Vega Trial
10. **Scientific title:** Evaluating the value of gym-based exercise versus usual care for young adults receiving antipsychotic medication: the Vega randomized controlled trial protocol
11. **Countries of recruitment:** Denmark
12. **Health conditions:** psychotic disorder or affective disorder
13. **Intervention(s)** Vega Exercise Community and Usual care.

**Vega Exercise Community:** Participants allocated to the Vega Exercise Community are offered one hour of tailored supervised, gym-based exercise training three times per week for a period of four months. In addition, participants are offered free-of-charge membership to the gym for six months and are invited to take part in training classes and use fitness equipment provided by the gym to regular members.

**Usual care**: Participants allocated to the usual care group will receive treatment as usual and be informed of the official physical activity guidelines as part of the information on group allocation. Moreover, they will be advised to continue their daily living, as they normally would do, not guiding them to other interventions neither preventing them to do so. In addition, participants in the usual care group will be given a subsidized membership including access to the Vega Exercise Community for four months after the 12 months follow up.

1. **Key Inclusion and Exclusion Criteria:** Patients aged 18-35 years who are currently treated with antipsychotic medication on a daily basis for at least one month (any dose and formulation) for the management of a psychotic disorder (F20-F29) or an affective disorder (F30-F39), who read and speak Danish will be eligible. Those who are advised against participating in exercise activities by their treating physician doctor (i.e., current injuries or instable somatic comorbidity) will be excluded.
2. **Study Type:** This is a multicenter pragmatic randomized (2:1) controlled superiority trial comparing the outcomes of the Vega Exercise Community versus usual care on personal recovery at 4-months.
   **Blinding:** The study staff who perform the assessments (baseline, four, six and 12 months), and the statistician will be blinded to group status. The instructors and participants will not be blinded to allocation due to the nature of the intervention.
   **Allocation:** Participants will be assigned to exercise training or usual care using an allocation ratio of 2:1 ratio in favor of exercise. Randomization will take place during the in-person visit after informed consent and baseline assessments have been completed. A computer-generated random number randomization list, stratified to center and diagnosis (F2/F3), with varying block sizes will ensure random allocation.
3. **Date of First Enrollment:** 25.10.2022
4. **Sample size:** The sample size was chosen to yield sufficient power for detection of a difference in change between the two groups of 5 points on the Questionnaire about the Process of Recovery (QPR) at 4 months with a standard deviation of 13.8 points which corresponds to an effect size of 0.36. Considering an allocation ratio of 2:1 in favor of the intervention group, a two-sided t-test with a significance level of 5% yields a power of 83% if 200 participants are included in the intervention group and 100 in the usual care group. We anticipate drop-out (i.e., participants that are lost to 4-month follow-up) of 20% and therefore a minimum of 375 participants is needed. However, given the limited research utilizing the QPR, we will aim to recruit 400 patients. This sample size will allow the detection of a clinically relevant difference between groups on our main secondary outcome, the Short-Form-12 Mental Component Summary (MCS), of 4 points at 4 months with a standard deviation of 12 points.
5. **Recruitment Status:** As of February 9^th^, 2023, 53 participants have been recruited.
6. **Primary Outcome(s):** The 15-item Questionnaire about the Process of Recovery (QPR) is the primary outcome and is used to measure personal recovery.
7. **Key Secondary Outcomes:** Secondary outcomes are related to health-related quality of life, behavioral symptoms, and metabolic health.
8. **Ethics Review:** Approval to conduct the study was received from The Ethics Committee for the Capital Region of Denmark (H-21079211) and the Danish Data Protection Agency (P-2022-78). All patients are informed verbally and in writing about the study and the rights of research participants. The Helsinki Declaration is followed for all aspects of the study including for data handling and protection of participant rights.
9. **Completion date:** Anticipated completion is Dec 2025.
10. **Summary Results:** Trial is ongoing. Results will be provided later.
11. **IPD sharing statement**:
    Plan to share IPD: Individual participant data (IPD) will be available for meta-analysis. Proposals should be directed to the sponsor. To gain access, data requestors will need to sign a data access agreement. If requests are received from third world countries then the Data Protection Act, Chapter V, will be followed.
