# Supplementary material for: Value of gym-based group exercise versus usual care for young adults receiving antipsychotic medication: study protocol for the multicenter randomized controlled Vega trial
Source: BMC Psychiatry. 2023 Aug 30;23:634. doi: 10.1186/s12888-023-05086-z (PMC10466717; doi:10.1186/s12888-023-05086-z)
Supplement: Supplementary file 3 — Additional file 3: The TIDieR (Template for Intervention Description and Replication) Checklist. [file 12888_2023_5086_MOESM3_ESM.docx]

**
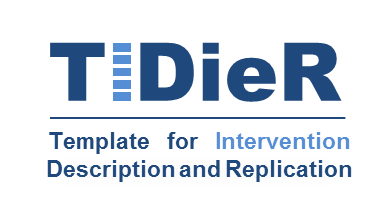
The TIDieR (Template for Intervention Description and Replication) Checklist*:**

Information to include when describing an intervention and the location of the information

| **Item number** | **Item** |
| --- | --- |
|  |  |
|  | **BRIEF NAME** |
| **1.** | **Provide the name or a phrase that describes the intervention.**  *Vega Exercise Community: 4 months of supervised group-based exercise 3 times a week in functional gyms* |
|  | **WHY** |
| **2.** | **Describe any rationale, theory, or goal of the elements essential to the intervention.**  *A gym-based exercise community offers social support and social inclusion in addition to training in social skills. Creation of an inclusive exercise community has the potential to reduce the symptoms (i.e., improve clinical recovery), and the experience of loneliness and internalized stigmatization which together may improve the participants’ experience of personal recovery.* |
|  | **WHAT** |
| **3.** | **Materials: Describe any physical or informational materials used in the intervention, including those provided to participants or used in intervention delivery or in training of intervention providers. Provide information on where the materials can be accessed (e.g. online appendix, URL).**  *Instructors in charge of the VEGA exercise community (intervention providers) received prior to an educational programme (see point 5: WHO PROVIDED) written material including following headlines:*   - *Introduction* - *Psychiatric diseases generally* - *Schizophrenia* - *Symptoms* - *Treatment* - *Living with schizophrenia* - *Myths and misunderstandings* - *Being a VEGA instructor* - *Recovery* - *Points of attention* - *Procedures regarding adverse events* - *VEGA exercise protocol (see point 4: procedures)* - *Adjustment and adaption of exercise protocol (see point 9: TAILORING)* - *Examples of individual VEGA programmes (see point 4: procedures)* - *Administrative task in relation to VEGA* |

| **4.** | **Procedures: Describe each of the procedures, activities, and/or processes used in the intervention, including any enabling or support activities.**  *A VEGA session will last 60 minutes, including a brief introduction of the daily program (which will be written on a whiteboard) and following headlines:*   - *Class warm-up (10-15 minutes including introduction)* - *Part A: Resistance training (10-20 minutes)* - *Part B: High Intensity Functional Training (HIFT) (10-20 minutes)* - *Cool down (5-10 minutes)*   *Warm-up will primarily target the physical demands in the following content in part A and B and include an active icebreaker or social teamexercise. Resistance training (part A) will focus on improving both strength and movement efficiency and have a combination of weightlifting and bodyweight exercises (see table below). HIFT (part B) will be provided as a “workout of the day” and incorporate all exercise modalities (see table below), with moderate to vigorous intensity. The combination of part A and B will strive to address all major muscle groups and the cardiovascular system in each VEGA session. Cool-down with focus on lowering arousal and conclude the session with low intensity mobility exercises.*   \| **Modality** \| **Description** \| **Example** \| \| --- \| --- \| --- \| \| Weightlifting \| *Movements that involve lifting or moving of an external object like barbell, dumbbells or kettlebells.* \| E.g., deadlifts, squats, press, push press, lunges. \| \| Bodyweight \| *Exercises that involve lifting or moving of a person’s own bodyweight* \| E.g., air-squats, push-ups, pull-ups, ring rows, burpees, sit-ups. \| \| Mono-structural \| *Traditional cardio exercises that are somewhat cyclical and allows for longer duration of performance.* \| E.g., rowing, running, skipping. \| \| Mobility \| *Exercises targeting the ability to move a joint through its full range of motion with maximum levels of physical control.* \| E.g. stretching, foam rolling, yoga positions. \|   *All sections are completed together as a group and some sessions will include team-workouts, where participants work together to complete the exercises. Part A and B will be presented in different arrangements as well as different time domains and include movement instructions before initiating the exercises (see table below)*   \| **Type of workouts** \| **Acronym** \| **Description** \| **Example** \| \| --- \| --- \| --- \| --- \| \| Rounds for quality \| RFQ \| *(Resistance training) One or more movements are performed after each other with a focus on quality. Tempo is relatively low with sufficient rest between rounds in order to emphasize correct technique.* \| 4 RFQ 8+8 Split squat with dumbbells 12 Ring rows \| \| Rounds for time \| RFT \| *(HIFT) Rounds of one or more exercises are performed as fast as possible. Exercises should be scaled so that participants move consistently well with correct technique even under higher stress. These types of workouts usually come with a timecap to give an idea of the intended stimulus.* \| 3 RFT 21 Kettlebell swings 15 Box Jumps 9 Burpees  Timecap: 12 minutes \| \| As many rounds/repetitions as possible \| AMRAP \| *(HIFT) One or more exercises are performed in a given window of time where the goal is to perform as many repetitions as possible. Exercises should be scaled so that participants move consistently well with correct technique even under higher stress.* \| AMRAP 15 200 Meters run 20 Dumbbells snatch 20 Push-ups 20 Ring rows \| \| Every minute on the minute \| EMOM \| *(HIFT) Interval-style training where a certain number of repetitions are performed within 1 minute. Exercises can switch each minute or stay the same.* \| EMOM 20 1. 8 Burpees 2. 10-15 Cal row 3. 50-70 Single/double under (skipping) 4. 10-15 Kettlebell goblet squat 5. Rest \|   *Each VEGA session will be facilitated centrally developed exercise program including the content of the session. 6 months of unique programs will be developed, and these programs will cycle the VEGA intervention period. All programs are based on a 5-day cycle that ensures a certain amount of variation in each VEGA session. (see table below)*   \|  \| **Resistance training (part A)** \| **HIFT (part B)** \| **Example + coaches notes** \| \| --- \| --- \| --- \| --- \| \| Day 1 \| Lower body \| Medium length (8-14 minutes) \| A: 4 RFQ  8 Barbell deadlift   B: AMRAP 10 10 Goblet squats 10 Push-ups 10 Ring rows ______________________________  Warm-up focus: Hips and posterior chain  A: Spend at least 10 minutes on teaching the deadlift and building up to a comfortable weight before they start. Do one set every 2^nd^ minute. Scale in weight or distance if technique is lacking.   B: Make sure to provide scaling options for each movement.  Goblet Squats can be scaled to air squats, pushups can be done on the knees or on a bench. \| \| Day 2 \| Bodyweight \| Longer duration (15-20 minutes) \| A: 3 RFQ  20 Step-ups 20 Sit-ups   B: EMOM 20 (:45 on/15: off) Min 1: Rowing machine Min 2: Jump rope Min 3: Burpees Min 4: Rest ______________________________  Warm-up focus: Heart rate  A: This is meant to be done with bodyweight. On the sit-ups participants should scale to a number that they can do in one set.   B: In this workout, participants work for 45 seconds followed by a 15 second transition on three different movements and then a minute rest. They go through this sequence five times, which in total concludes 20 minutes of work. Pace should be sustainable for the 45 seconds, scale accordingly. \| \| Day 3 \| Upper body \| Medium length (8-14 minutes) \| A: 4 RFQ 10 Dumbbell bench press 10+10 One arm dumbbell rows  B: 4 RFT 15 Kettlebell swings 15 Box Jumps Timecap: 10 minutes  ______________________________  Warm-up focus: Upper body  A: Team up two and two for a bench. dumbbell rows can be done standing at a box if there are not enough benches available. Each set should take about 3 minutes allowing for some at least 1 minute of rest between sets.   B: Make sure to provide scaling options, especially for the box jumps where participants can either decrease the jumping distance or do step-ups instead. Fatigue will add up during the workout, so some might need to scale after the first round or two. \| \| Day 4 \| Lower Body \| Shorter intervals (2-7 minutes) \| A: 4 RFQ  8 Barbell back squat   B: 3 x AMRAP 3 / 1 min rest 1-2-3-4-5…. Push-ups Dumbbell snatch ___________________________________  Warm-up focus: Squat positions   A: Team up in groups of two/three per rack. Spend at least 8 minutes on teaching the squat so they are comfortable with this movement pattern. Do one set every 2^nd^ minute. Scale in weight if technique is lacking.  B: Help participants scale these movements, so that they can keep both high pace and good movement quality through each interval. Scaling can also be done as the workouts proceeds. \| \| Day 5 \| Upper body \| Longer duration (15-20 minutes) \| A: 5 RFQ  10-10-8-8-6 Barbell shoulder Press   B: AMRAP 15 200 meters run 20 Goblet lunges 20 Ring rows ______________________________________________  Warm-up focus: Shoulders   A: Team up in groups of two/three per rack. Spend at least 8 minutes on instruction with an empty barbell before participants go for the working sets. Weight should start conservative and increase as the reps go down.   B: The priority for this workout is to move at a steady pace for 15 minutes. Running can be scaled to rowing if participants are not comfortable with being outside. Difficulty on the lunges and rows should be a level where they can do at least 10 reps. \|   *To support development of exercise self-efficacy, instructors and ideally also peer mentors will meet with each participant individually before initiation of the exercise program.* During this meeting, the participant will complete an exercise self-efficacy questionnaire (developed for the study specifically), exploring previous exercise experience and preferences on how she/he is likely to prefer being coached and receive feedback*. This meeting will furthermore include discussion/development of individual goals. Furthermore, VEGA instructors are taught different methods of tailoring the workouts and exercises to all fitness levels (see 9, TAILORING) and focusing on success to promote exercise self-efficacy.*  *During the exercise period, short text messages will be sent by the instructor to all participants the day before each VEGA session (and/or in the morning in the case of classes scheduled in the afternoon), encouraging participants to show up. Participants will be invited to a group on social media administrated by a member of the research team and reserved for intervention group members and instructors.*  *Lastly all participants will be invited to participate in semi-annually Obstacle Course Racings which will facilitate as common goals between the participants.* |
| --- | --- | --- | --- | --- | --- | --- | --- | --- | --- | --- | --- | --- | --- | --- | --- | --- | --- | --- | --- | --- | --- | --- | --- | --- | --- | --- | --- | --- | --- | --- | --- | --- | --- | --- | --- | --- | --- | --- | --- | --- | --- | --- | --- | --- | --- | --- | --- | --- | --- | --- | --- | --- | --- | --- | --- | --- | --- | --- | --- | --- |
|  | **WHO PROVIDED** |
| **5.** | **For each category of intervention provider (e.g. psychologist, nursing assistant), describe their expertise, background and any specific training given.**  *The instructors are expected to have experience as a fitness instructor, but no other formal qualifications are required. Instructors will be recruited through commercial functional gyms hosting the VEGA intervention. All instructors will be approved by the VEGA research team.*  *The instructors will be required to have completed an educational programme. The programme was conducted using a twostep qualitative process including interviews with key informants and focus groups with relevant stakeholders. A scientific paper regarding the development of the educational programme will be conducted (not yet published).*  *Prior to participating in the educational programme, VEGA instructors will receive written material (see point 3: materials).*  *The educational programme (1 day)*   - *Presentation and rational regarding the VEGA trial* - *Interview and e-learning by psychiatrist regarding schizophrenia* - *Interview story from psychiatric nurse working in OPUS (outpatient clinic)* - *E-learning with psychiatric nurse and recovery researcher regarding recovery* - *Interview story from former patient with schizophrenia* - *Delivering VEGA exercise community* - *Conducting start-up exercise self-efficacy meetings (see point 4: procedures)* - *Procedures regarding adverse events* - *Administrative VEGA work tasks* - *Evaluation, Q&A and rounding up* |
|  | **HOW** |
| **6.** | **Describe the modes of delivery (e.g. face-to-face or by some other mechanism, such as internet or telephone) of the intervention and whether it was provided individually or in a group.**  *The intervention will be delivered as face-to-face supervised group-based exercise facilitate by two instructors (see point 5: who provided) and ideally supplemented by a peer supporter. The peer-supporter will not attend all VEGA sessions but serve as equally member of the exercise community but with extended knowledge of living with a psychotic disorder and have completed a peer course provided by local psychiatric hospitals.* |
|  | **WHERE** |
| **7.** | **Describe the type(s) of location(s) where the intervention occurred, including any necessary infrastructure or relevant features.**  *The intervention will be delivered at following addresses.*   \| *CrossFit North 579*  *Hjulmagervej 11*  *9000 Aalborg*  *Denmark* \| *ARCA, Værkstedet*  *Kastanie Allé 20*  *2720 København*  *Denmark* \| *ARCA Smedjen*  *Sigurdsgade 37*  *2200 København*  *Denmark* \| *ARCA, Fundamentet*  *Helsingforsgade 23*  *8200 Aarhus*  *Denmark* \| \| --- \| --- \| --- \| --- \|   *Public transportations are available within few hundreds meter from each intervention site.*  *The VEGA exercise community will take place in training facilities designed for functional training. Facilities have the possibility for privacy (can be secluded) and are equipped with rubber floors and pullup-bar for bodyweight movements, as well as barbells, dumbbells, kettlebells, medicine balls, rowing machines, etc. running routes of 200 and 400 meters are also provided.* *Dressing rooms, lockers and showers are also accessible. Lastly all facilities have a “social corner” where participants can hang out before and after the session. (see pictures below for example of facilities)*     \| *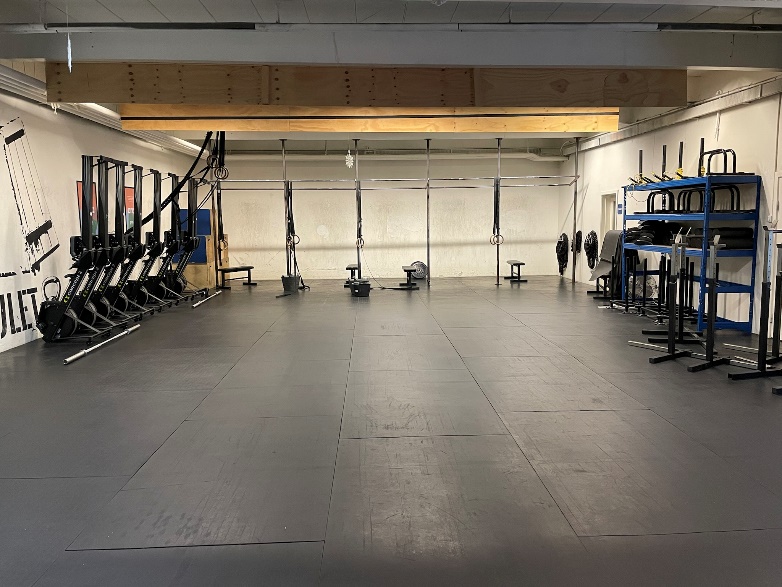* \| *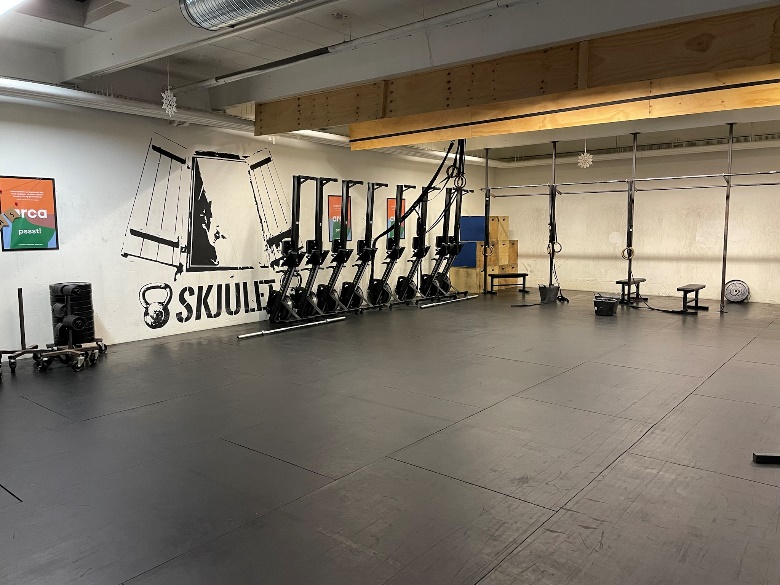* \| *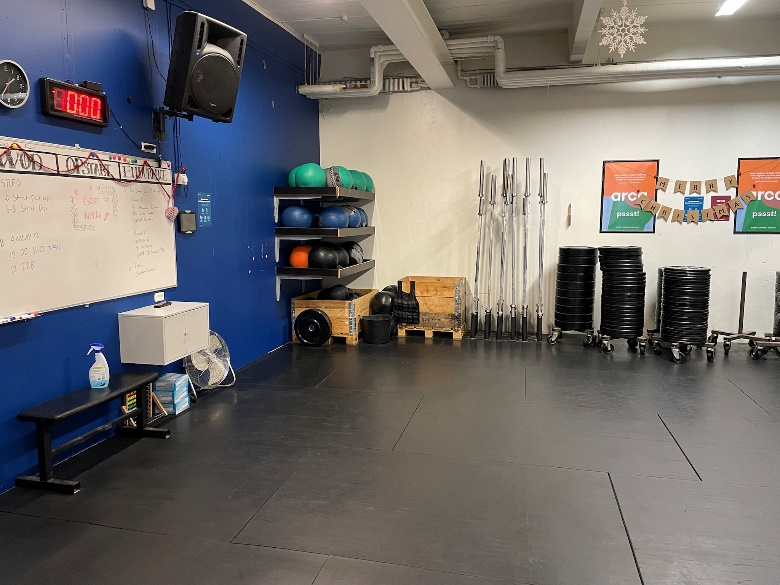* \| \| --- \| --- \| --- \| |
|  | **WHEN and HOW MUCH** |
| **8.** | **Describe the number of times the intervention was delivered and over what period of time including the number of sessions, their schedule, and their duration, intensity or dose.**  *Participants allocated to the Vega Exercise Community (i.e., intervention group) will be offered one hour of tailored supervised, gym-based exercise training three times per week for a period of four months. In addition, participants are offered free-of-charge membership to the gym for six months and are invited to take part in training classes and use fitness equipment provided by the gym to regular members.*  *Further information regarding the intervention see point 4: procedures.* |
|  | **TAILORING** |
| **9.** | **If the intervention was planned to be personalised, titrated or adapted, then describe what, why, when, and how.**  *To ensure both quality of movement and preservation of the intended stimuli of the workout, the instructor will provide scaling options for each exercise. This can be done by altering the load in either weightlifting or bodyweight movements, or by decreasing the range of motion with a given movement. Furthermore, there is an option of scaling the complexity of an exercise by changing it to another. The workout itself can also be altered by reducing the number of repetitions or rounds/sets. Scaling will be done on an individual basis according to the participants experience and abilities. The instructor will present these modifications to make the workout fit individual rather than a view of not being capable.*  *In addition, each exercise or activity can be adapted to the participants psychological and social capacities on the day, e.g., by reducing the social interaction in group-based exercises or altered exercise possibility if symptoms somehow prevent the participant from participating.* |
|  | **MODIFICATIONS** |
| **10.^ǂ^** | **If the intervention was modified during the course of the study, describe the changes (what, why, when, and how).**  *Not applicable* |
|  | **HOW WELL** |
| **11.** | **Planned: If intervention adherence or fidelity was assessed, describe how and by whom, and if any strategies were used to maintain or improve fidelity, describe them**.  *Adherence will be monitored by VEGA instructors after each session using an app (Holdsport.dk). Further, the use of the subsidized membership (i.e., participation in other exercise classes) will be collected from the fitness centres’ registration system.*  *Fidelity will be measured by collecting data on intensity, duration, structure, and organization of the session. Specifically, the duration, structure (i.e., warm-up, part A, part B and cooldown) and organization (i.e., presence of two instructors and peer-supporter) will be reported by the instructors in all sessions using an app (Holdsport.dk). The exercise intensity will be assessed using heart rate monitors worn by the participants and self-reported using the Borg Scale of perceived exertion in selected sessions.*  *Members of the research teams will occasionally overview the VEGA sessions to control the registration of adherence and compliance regarding fidelity from the instructors.* |
| **12.^ǂ^** | **Actual: If intervention adherence or fidelity was assessed, describe the extent to which the intervention was delivered as planned.**  *Not applicable* |

** **Authors** - use N/A if an item is not applicable for the intervention being described. **Reviewers** – use ‘?’ if information about the element is not reported/not sufficiently reported.

† If the information is not provided in the primary paper, give details of where this information is available. This may include locations such as a published protocol or other published papers (provide citation details) or a website (provide the URL).

ǂ If completing the TIDieR checklist for a protocol, these items are not relevant to the protocol and cannot be described until the study is complete.

* We strongly recommend using this checklist in conjunction with the TIDieR guide (see *BMJ* 2014;348:g1687) which contains an explanation and elaboration for each item.

* The focus of TIDieR is on reporting details of the intervention elements (and where relevant, comparison elements) of a study. Other elements and methodological features of studies are covered by other reporting statements and checklists and have not been duplicated as part of the TIDieR checklist. When a **randomised trial** is being reported, the TIDieR checklist should be used in conjunction with the CONSORT statement (see [www.consort-statement.org](http://www.consort-statement.org)) as an extension of **Item 5 of the CONSORT 2010 Statement.** When a **clinical trial** **protocol** is being reported, the TIDieR checklist should be used in conjunction with the SPIRIT statement as an extension of **Item 11 of the SPIRIT 2013 Statement** (see [www.spirit-statement.org](http://www.spirit-statement.org)). For alternate study designs, TIDieR can be used in conjunction with the appropriate checklist for that study design (see [www.equator-network.org](http://www.equator-network.org)).
