# Supplementary figures and images for: Value of gym-based group exercise versus usual care for young adults receiving antipsychotic medication: study protocol for the multicenter randomized controlled Vega trial
Source: BMC Psychiatry. 2023 Aug 30;23:634. doi: 10.1186/s12888-023-05086-z (PMC10466717; doi:10.1186/s12888-023-05086-z)

**Supplementary file 4**: Program theory


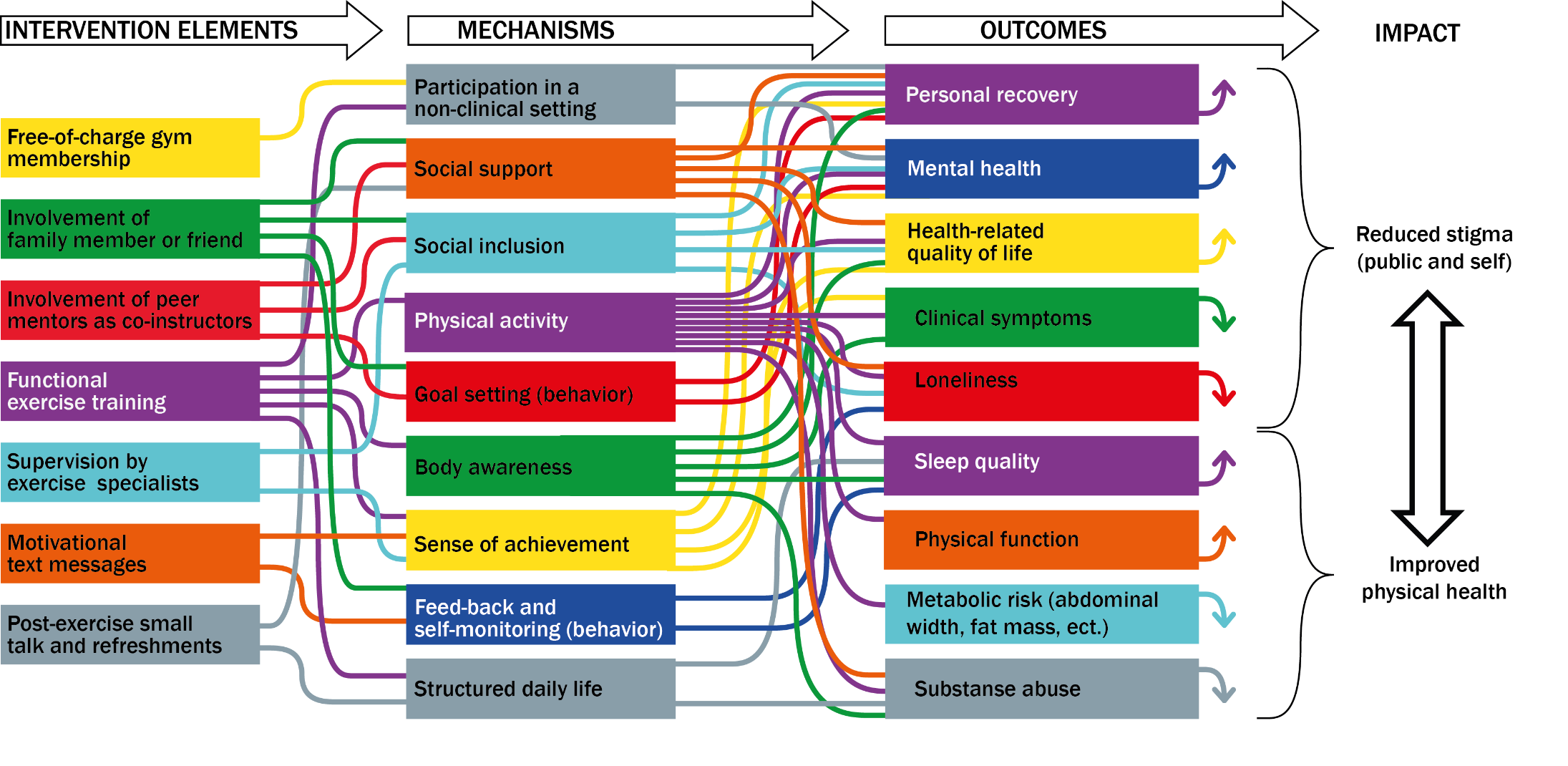

Supplement: Supplementary file 4 — Additional file 4: Vega Program theory. [file 12888_2023_5086_MOESM4_ESM.docx]
